# Highlights of the 16th annual scientific sessions of the Society for Cardiovascular Magnetic Resonance

**DOI:** 10.1186/1532-429X-15-60

**Published:** 2013-07-19

**Authors:** John-Paul Carpenter, Amit R Patel, Juliano Lara Fernandes

**Affiliations:** 1 Royal Brompton Hospital, London, UK; 2 University of Chicago, Chicago, USA; 3 University of Campinas, Campinas, Brazil

**Keywords:** Cardiovascular magnetic resonance, Heart

## Abstract

The 16th Annual Scientific Sessions of the Society for Cardiovascular Magnetic Resonance (SCMR) took place in San Francisco, USA at the end of January 2013. With a faculty of experts from across the world, this congress provided a wealth of insight into cutting-edge research and technological development. This review article intends to provide a highlight of what represented the most significant advances in the field of cardiovascular magnetic resonance (CMR) during this year’s meeting.

## Introduction

This year’s SCMR Annual Scientific Sessions were attended by a record number of 1427participants. The number of abstracts presented during the meeting also grew significantly from previous editions with a total of 602 abstracts being shown. Many of these new abstracts were introduced during the SCMR/International Society for Magnetic Resonance in Medicine (ISMRM) Jointly Sponsored Workshop “New Horizons in High-field Cardiovascular Imaging: Promises and Progress” as well as in the new ePosterGallery.

In this article, we have tried to cover most of the themes presented during the five days of the event summarizing the topics into technical/imaging methods and clinical applications. Throughout the meeting, the SCMR Program Committee ensured that all of the major areas of interest were covered with an invited lecture session to complement the scientific abstract presentations. All abstracts presented during the meeting can be accessed in the special supplement of the Journal of Cardiovascular Magnetic Resonance [1]. Many of the invited lectures and oral abstract sessions were recorded and are available at http://www.scmr.org.

## Review

Details of the SCMR Gold Medal award for outstanding contribution to the field of CMR (awarded to Professor Stefan Neubauer), the best oral presentations and abstract award winners for both SCMR annual scientific sessions and the SCMR/ISMRM workshop are given in Tables 1 and 2. The winning authors of the Gerald Pohost award for best Journal of Cardiovascular Magnetic Resonance (JCMR) manuscript in 2012 are shown in Table 3.

**Table 1:** 16th SCMR annual scientific sessions – awards

**Award**	**Recipient**	**Institution**	**Subject**
SCMR Gold Medal	Professor Stefan Neubauer	Oxford University	For outstanding contribution to the field of CMR
Early career award – basic science	Behzad Sharif	Cedars-Sinai Medical Center	Eliminating dark-rim artifacts in first-pass myocardial perfusion imaging (O3)
Early career award – translational	James Harrison	King’sCollege London	Magnetic resonance imaging of acute and chronic atrial ablation injury – a histological validation study (O18)
Early career award – clinical	Saira Dass	The John Radcliffe Hospital	Patients with dilated cardiomyopathy (DCM) have appropriate myocardial oxygenation response to vasodilator stress (O68)
Moderated poster session 1	Kathryn Broadhouse	Imperial College London	Quantification of aortic pulse wave velocity in preterm infants using 4D phase contrast MRI (M7)
Moderated postersession 2	Daniel Kuetting	Universityof Bonn	Assessment of cardiac dyssynchrony: a comparison of velocity encoded imaging and feature tracking analysis (M11)
Best technologist abstract	Celia O’Meara	UniversityCollege London Hospitals	Initial experience of imaging cardiac sarcoidosis using hybrid PET-MR – a technologist’s case study (T1)
Best SCMR web case of the year	Rob Huggett	Russells Hall Hospital, UK	Post pericardiectomy for constriction – a late complication

**Table 2:** SCMR/ISMRM workshop abstract awards

**Award**	**Recipient**	**Institution**	**Manuscript title**
Best Oral Abstract	Gabriel Camargo	Clinica de Diagnostico por Imagem, Rio de Janeiro, Brazil	Myocardial Iron Quantification Using Modified Look-Locker Inversion Recovery (MOLLI) T1 Mapping at 3 Tesla
Best Poster	Johannes Krug	Otto-von-Guericke University of Magdeburg	Improved ECG Based Gating in Ultra High Field Cardiac MRI Using an Independent Component Analysis Approach

**Table 3:** Gerald Pohost award for best Journal of Cardiovascular Magnetic Resonance (JCMR) manuscript in 2012

**Award**	**Recipient**	**Institution**	**Manuscript title**
First place	Jurg Schwitter	University Hospital, Lausanne	Superior diagnostic performance of perfusion-cardiovascular magnetic resonance versus SPECT to detect coronary artery disease: The secondary endpoints of the multicenter multivendor MR-IMPACT II (Magnetic Resonance Imaging for Myocardial Perfusion Assessment in Coronary Artery Disease Trial). [2]
Second place	Peter Kellman	National Institutes of Health (NIH)	Extracellular volume fraction mapping in the myocardium, part 1: evaluation of an automated method. [3]
Third place	Choukri Mekkaoui	Harvard Medical School	Fiber architecture in remodeled myocardium revealed with a quantitative diffusion CMR tractography framework and histological validation. [4]

## Technical advances/imaging methods

**Figure 1:**
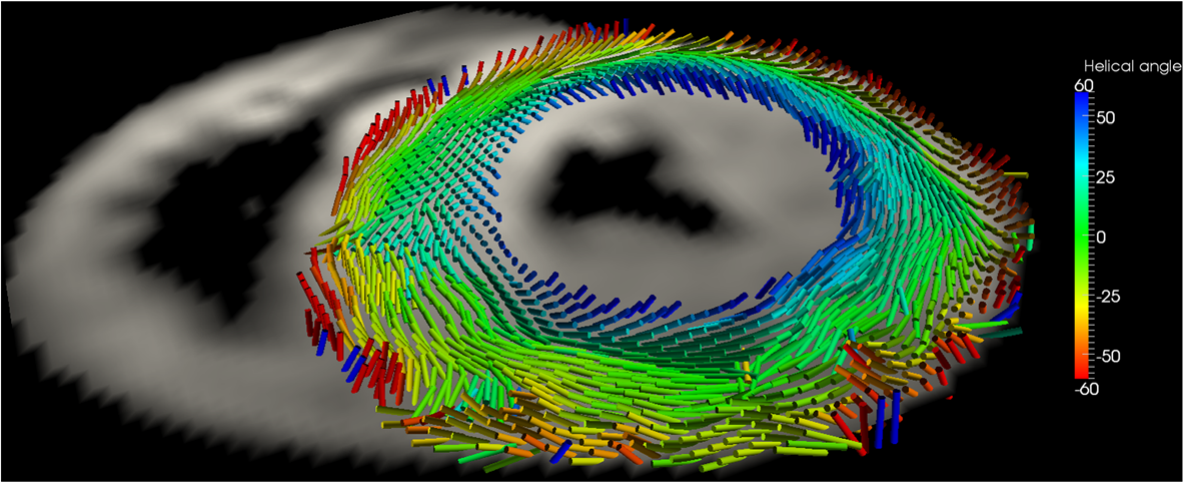
**Diffusion tensor imaging in the basal short-axis plane in a patient with hypertrophic cardiomyopathy.** The acquisition used a diffusion-weighted stimulated-echo single-shot echo-planar-imaging sequence during multiple breath holds. The tensor’s main eigenvector, which follows the orientation of the myocytes, is represented by cylindrical glyphs, colour coded according to the helix angle: blue right-handed, green circumferential, red left-handed (Image courtesy of Pedro Ferreira and Dudley Pennell).

This year’s SCMR/ISMRM pre-Sessions workshop focused on the development of high-field CMR, an increasing area of interest as scanners move from 1.5 T to 3.0 T and even 7.0 T systems [5,6]. Challenging problems with high field strengths currently involve the development of specific transmit/receive coils to allow assessment of 23Na-MRI as well as CMR-spectroscopy, new imaging sequences to take advantage of the increase in signal-to-noise ratio (SNR) as well as better ways to handle ECG gating. In that regard, Ferreira et al. presented a new free-breathing navigator sequence to assess cardiac diffusion tensor imaging (DTI) at 3 T to characterize myocardial fiber disarray patterns as has recently been published [7-9] while Teh et al. demonstrated a new accelerated diffusion-weighted fast spin echo sequence that resulted in an 8-fold increase in acquisition time in a mouse model using 9.4 T scanning [10]. DTI was the subject of one of three plenary lectures delivered by Dudley Pennell at the opening of the main meeting (http://scmr.org/Education/CMR-online-video-on-demand-lectures/3072/3248/3252.html) – Figure 1. As mentioned above, ECG gating becomes more difficult with increasing field strength due to magnetohydrodynamic effects of the higher magnetic fields. To circumvent that problem, Krug et al. demonstrated the problems encountered with regular vectorcardiograms at 3.0 and 7.0 T proposing an independent component analysis alternative [11] and Tse et al. presented a new 3D-QRS method using 12-lead ECG gating inside the magnet to allow for real-time gating at 3.0 T in patients with complex heart rates such as premature ventricular beats, atrial fibrillation and significant beat-to-beat variations [12].

### Increasing spatial/temporal resolution

Many advances presented this year dealt with technical developments with the objective of increasing the spatial and/or temporal resolution of CMR images as well as decreasing image acquisition time. One major focus was how to implement new methods in order to acquire real-time non triggered images allowing much more simple and robust imaging as well as permitting the use of CMR to guide percutaneous interventions [13]. Aneja et al. demonstrated that real-time CMR using non-optimized sequences with lower spatial and temporal resolution already derives similar results to traditional breath-held images in patients with regular cardiac rhythm [14]. Although this approach was limited to a small number of patients and using standard sequences only, the principles of real-time imaging are rapidly gaining acceptance as CMR increases its clinical utility.

With that objective in mind, an increasing number of abstracts focused on new sequences that take advantage of undersampling the k-space with spatiotemporal modeling such as compressed sensing [15] to increase current resolution limits. Using sparse and incoherent sampling with multiple iterative reconstructions Schmidt et al. showed that these new techniques provide high spatial (2.4 × 1.7 × 6.0 mm) as well as temporal resolution (33 ms) within 1 heartbeat acquisition in real time [16]. Introducing a new regional sparsity reconstruction method, Chen et al. also demonstrated the feasibility of compressed sensing for obtaining in vivo perfusion images, allowing for increase in ventricular coverage or spatial resolution as previous techniques have shown [17,18].

Non-cartesian acquisitions have also seen a gain in use and moved from the preliminary work in coronary artery imaging to other applications in CMR [19]. Sharif et al. used an ungated radial acquisition method to acquire first-pass perfusion images simultaneously combined with cine images for assessment of perfusion and wall motion within the same scan [20]. Yang et al. also used a combination of spiral trajectories with compressed sensing to obtain full ventricular coverage during first pass perfusion with the acquisition of 8 slices in 480 ms with 1.75 × 1.75 mm in-plane resolution allowing for heart rates up to 125 beats per minute during stress imaging [21]. In another abstract by Petersson et al. the authors demonstrated the use of spiral k-space trajectories to acquire 4D flow stacks with a significant 62% reduction in scan time while maintaining similar image quality compared to traditional readout methods [22].

### Improving artifacts

**Figure 2:**
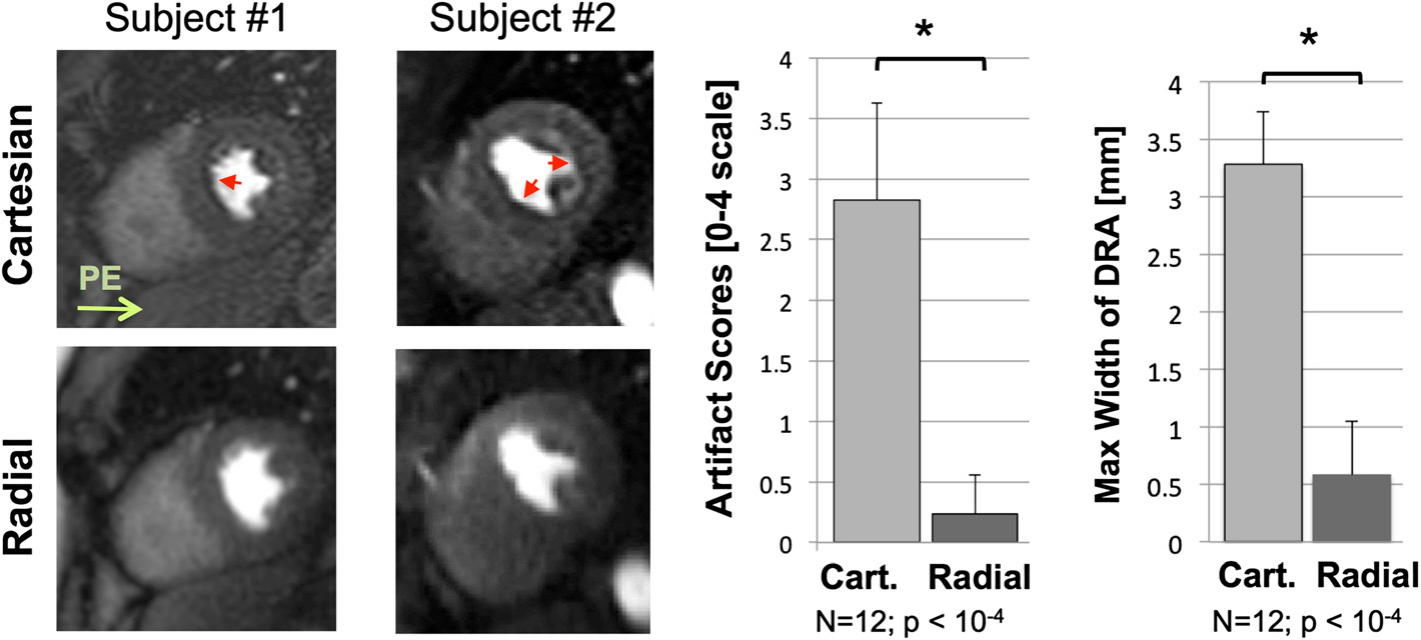
**Elimination of dark rim artifact on first-pass perfusion imaging using radial k-space trajectory.** The left hand panel shows examples of first pass perfusion images in two subjects. The artifact seen in the phase encode (PE) direction with Cartesian trajectory (top row) is eliminated with radial k-space filling (bottom row). The histograms reveal a significant reduction in artifact scores (on a scale of 1–4) and maximum width of dark rim artifacts (DRA) with the radial technique (Sharif et al., abstract O3).

Another highlight of the meeting was in the developments of new methods to overcome old problems in CMR such as dark rim artifacts, off-resonance effects and banding [23]. The Early Career Award in Basic Sciences was awarded to Sharif et al. for an abstract that proposed to reduce dark-rim artifacts during first-pass perfusion by using a single-shot radial sequence in comparison to the routine Cartesian technique with a significant decrease in both artifact score as well as in its maximum width (Figure 2) [24]. Problems with 3 T MRI were also addressed by Wu et al. who proposed a shorter TR for SSFP cine imaging through a reduced RF pulse duration and readout showing that the new approach resulted in images more robust to field in homogeneity at this higher field strength with minimum reduction in spatial resolution while allowing for elimination of frequency scouts and more rapid breath-hold times [25]. Another way to improve susceptibility artifacts without the need to use time consuming frequency adjustments was presented by Rothstein et al. who proposed a new 3D shimming algorithm which resulted in similar results for volumes and function evaluation keeping the same levels of banding artifacts as frequency offsets but with a more simplified workflow [26].

### T1 Mapping sequence developments

With the rapidly increasing body of new clinical data on T1 mapping, more understanding of the fundamental principles of the sequences used to generate these maps is essential [27]. A comprehensive series of lectures on T1 mapping in the heart was presented by Peter Kellman, Martin Ugander, Erica Dall’ Armellina and Andrew Flett (http://scmr.org/Education/CMR-online-video-on-demand-lectures/3072/3151/3154.html).This provided a clear explanation of the pros and cons of each of the different sequences together with the importance of both accuracy and precision of measurement. The potential clinical applications of T1 mapping including the measurement of extracellular volume fraction to visualize the extent and severity of abnormalities within the myocardium were outlined in detail.

Almost 10% of the submitted abstracts for the meeting involved T1 mapping. Many sessions discussed the current use of T1 maps and the need to standardize and optimize the acquisition techniques to pre- and post-contrast maps, the use of bolus versus continuous infusion of gadolinium as well as the different approaches to heart rate variability and types of sequences. Fitts et al. presented a recent arrhythmia-insensitive rapid T1 mapping sequence to overcome some of the limitations of the most widely used modified Look-Locker inversion recovery (MOLLI) techniques [28]. The sequence is based on saturation recovery magnetization and uses centric k-space ordering resulting in smaller coefficients of variation and higher T1 values in patients with high heart rates or arrhythmias. In another study using phantoms, Slavin et al. also showed that using a saturation recovery pulse but with a balanced SSFP readout they could find more accurate T1 values compared to MOLLI due to its insensitivity to beat-to-beat variations and lack of T2 dependence [29]. In a comparison of different sequences including the classical MOLLI sequence, a shortened (sh)MOLLI acquisition with 9 heartbeats [30], a 3′5 MOLLI and a saturation recovery single shot acquisition sequence (SASHA) the authors reported that the 3′5 MOLLI sequence was more closely associated to the original MOLLI T1 values compared to the two other sequences while the shMOLLI technique underestimated T1 values by 13% and the SASHA sequence overestimated the T1 values by 17%. Finally, a 3D technique acquired with free breathing was also presented by Henningsson et al. with the average scan time of 9:37 minutes to cover the whole left ventricle with a resolution of 1.5 × 1.5 × 8 mm^3^ using SASHA resulting in an increase in SNR compared to 2D-SASHA images with similar T1 values [31].

### Developments in vascular imaging

The development of rapid prototyping (or 3D printing) has allowed highly accurate models of vascular structures to be generated [32]. Acevedo-Bolton et al. described how complex intracranial aneurysms imaged with magnetic resonance could be recreated in order to test the effectiveness of flow-diverting stents [33]. This work not only enables the operator to gain valuable experience on a ‘dry run’ but allows assessment of the pre- and post-procedure flow dynamics using 4D flow.

Three other oral presentations described the utility of the quiescent-interval single shot or ‘QISS’ sequence for non-contrast MR angiography. The technique (with various alterations including radial vs. Cartesian geometry k-space acquisition) shows excellent performance and correlates very well with contrast exams in patients with peripheral arterial disease. These angiographic techniques have the potential to be combined with more functional assessment (such as rest and stress calf perfusion) to assess ischemia and response to treatment [34]. Clement-Guinaudeauet al presented data on a calf muscle perfusion index which correlated very well with peak walking time and claudication onset time [35]. Using a simple MR-compatible device to allow exercise stress in the scanner bore, this has useful potential as a surrogate endpoint for clinical trials.

## Implementation of CMR into routine clinical practice

In addition to many technical developments, this year’s SCMR also featured several presentations showing the central role of CMR in the management of the cardiovascular patient. One of the strengths of this year’s conference was the introductory talk given by an expert in the field at the start of each of the oral abstract sessions. Raymond Kwong succinctly summarized the available evidence from the last 25 years on how the development of CMR has helped diagnosis, determine prognosis and provide an impact on diagnostic thinking, therapeutic actions and patient outcomes. Anya Wagner presented the most recent results of the EuroCMR registry covering 27,000 patients from 57 European centers [36]. This has cemented CMR at the center of diagnostic imaging in Europe, proving that it is feasible and safe with 62% of the scans performed having an impact on patient management, the most common indication being for risk stratification in coronary artery disease. Florian Andre presented data from a cohort of more than a 1000 patients showing that measuring left ventricular ejection fraction by CMR reclassified many patients into different risk groups when compared to echocardiography [37]. Others have shown the potential clinical impact of more accurate measurement of ejection fraction by CMR on selecting patients who might benefit from defibrillator implantation [38]. Recently, it has been demonstrated that the addition of late gadolinium enhancement CMR adds further prognostic value over echocardiography alone [39]. Additionally, it is becoming increasingly recognized that using CMR in a cost-effective way can provide potential savings for healthcare providers [40].

### Ischemic heart disease

With the publication of CE-MARC [41] and MR-IMPACT 2 [2] vasodilator stress CMR has been shown in prospectively designed studies to be a reasonable alternative to stress single-photon emission computed tomography (SPECT) for the detection of significant coronary artery disease. It has additionally been shown that performing stress CMR to identify patients who benefit from coronary angiography offers significant savings when compared to directly performing coronary angiography. John Greenwood adds to this by presenting new data from the CE-MARC trial suggesting that strategies the utilize stress CMR first followed by invasive coronary angiography if the CMR study is abnormal or inconclusive may be more cost-effective when compared to a SPECT first strategy [42]. Similarly, Sebastian Kelle showed the cost-effectiveness of strategies for the assessment of stable coronary artery disease that utilize dobutamine stress to identify patients who might benefit from a coronary angiogram rather than sending all patients directly to cardiac catheterization [43]. Using a Markov analysis, Steffen Petersen presented data suggesting that the cost-effectiveness of a diagnostic strategy was dependent on the pretest probability of having coronary artery disease. His analysis suggested stress CMR strategies were most cost effective in patients with a higher pretest probability and that cardiac computed tomography strategies were more cost-effective in patients with a low to intermediate pretest probability [44].

**Figure 3:**
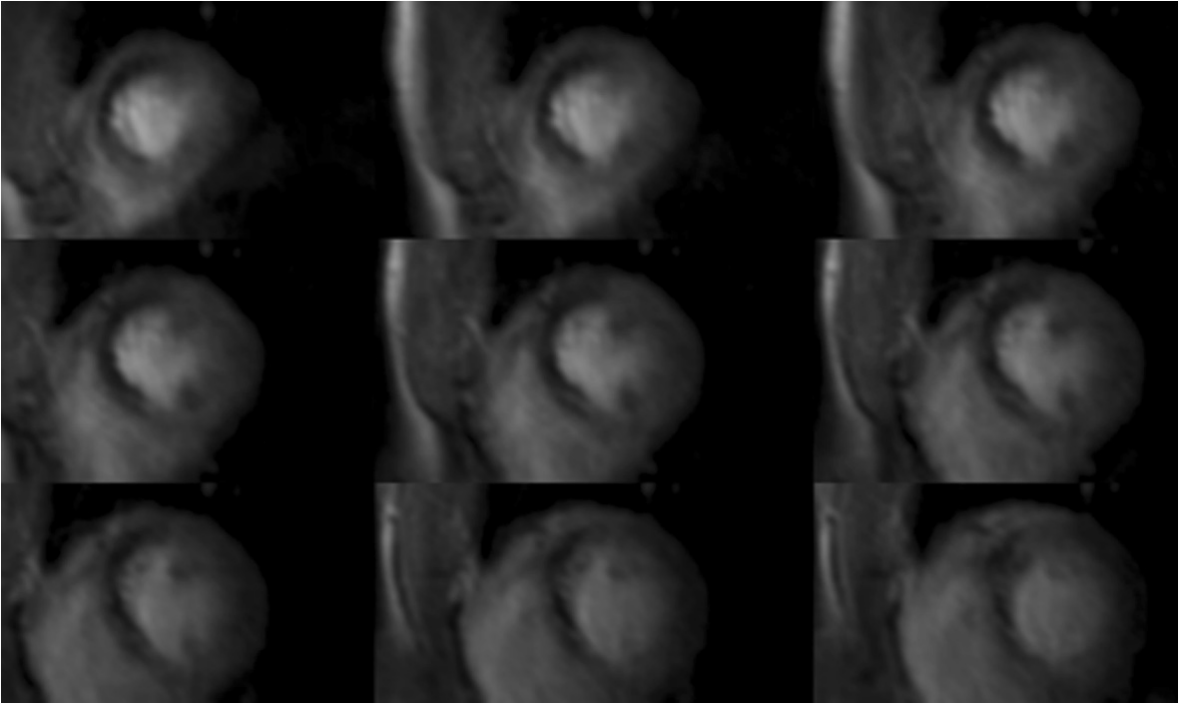
**3D CMR perfusion scan during adenosine infusion showing an inducible perfusion defect in the anterior and anteroseptal walls extending from the base to the apex.** Invasive coronary angiography identified a high-grade stenosis in the left anterior descending artery (Manka et al., abstract O103).

An important limitation of stress CMR is the incomplete coverage of the left ventricle and may prevent adequate assessment of ischemic burden. Sven Plein compared the ischemic and scar burden as determined by a standard clinical stress CMR technique to SPECT [45]. He found that CMR measures significantly less scar but more ischemia than SPECT possibly due to the incomplete coverage of perfusion CMR. In a prospective, multicenter trial, Robert Manka showed that whole heart, three-dimensional myocardial perfusion imaging (Figure 3) correlated highly with fractional flow reserve [46].It remains to be seen whether such a technique will improve the quantification of ischemic burden and whether the burden of ischemia as measured by CMR can identify patients who will benefit from revascularization in addition to aggressive medical therapy alone.

### Non-ischemic heart disease

The role of CMR in the assessment and risk stratification of non-ischemic heart disease is well established [47]. Currently available data detail the ability of CMR to risk stratify patients with a variety of non-ischemic heart diseases such as dilated cardiomyopathy, myocardial siderosis, and hypertrophic cardiomyopathy (HCM) [48]. However, large outcome studies remain challenging. Ismail et al. presented data on 711 consecutive patients with HCM with a total of 2852 patient-years of follow-up [49]. Although the amount of fibrosis was a significant univariate predictor of sudden cardiac death risk, on multivariate analysis, only left ventricular ejection fraction remained a significant predictor. The understanding of the HCM phenotype is becoming ever more complex [48,50]. Deva explained the incremental predictive value of inferoseptal crypts [51] and Flett proposed a previously unrecognized pattern of relative apical hypertrophy which may be an early form of HCM as the group found many features of HCM in patients with unexplained T-wave inversion [52]. Christopher Kramer unveiled the details of a proposed large scale, multiparametric, multicenter, observational registry with the goal of better defining the role of CMR in the risk stratification of the HCM patient.

As previously mentioned, T1 mapping featured highly throughout the sessions. Sado et al. presented findings of low pre-contrast T1 values in Anderson-Fabry disease and iron overload which may help to define pre-clinical phenotypes more accurately [53]. Whereas the assessment of diastolic function has traditionally been cumbersome and time-consuming with CMR, Simpson et al. have developed a new high spatial and temporal resolution tissue phase contrast technique which allows much shorter acquisitions and brings this nearer to clinical prime-time [54]. In an invited lecture, Michael Markl reviewed promising techniques such as tissue phase mapping with respiratory navigator gating, 2-dimensional and 3-dimensional Displacement Encoding with Stimulated Echoes (DENSE), and strain encoded imaging (SENC) for assessing myocardial mechanics and quantifying the various components of myocardial strain (http://scmr.org/Education/CMR-online-video-on-demand-lectures/3072/3157/3160.html). During the same session, Alicia Maceira, Amit Patel, and Sophie Mavrogeni suggested that CMR with late gadolinium enhancement imaging plays an important role for the detection of cardiac involvement and risk stratification of patients with systemic disorders such as amyloidosis, vasculitis, and other rheumatologic disorders such as systemic lupus erythematosis, sarcoidosis, rheumatoid arthritis, and systemic sclerosis (http://scmr.org/Education/CMR-online-video-on-demand-lectures/3072/3157/3161.html).

### CMR and electrophysiology

Francisco Leyva reviewed the role of CMR for identifying patients that may benefit from defibrillator implantation and resynchronization therapy (http://scmr.org/Education/CMR-online-video-on-demand-lectures/3072/3163/3164.html). Several investigators have shown that the extent of late gadolinium enhancement is strongly associated with need for future ICD therapy [55]. The burden of late gadolinium enhancement is also associated with likelihood of responding to resynchronization therapy; in fact, using late gadolinium enhancement images to guide left ventricular lead placement improves outcomes following biventricular pacing [56].

The continued development of real-time imaging for interventional CMR procedures is an important area of research. Tse et al presented preliminary animal data on a voltage-based electroanatomic tracking system which can be used to measure intracardiac electrograms both in and out of the scanner bore, including during imaging sequences. Radiofrequency signals from patches on the skin allow the operator to track the navigation of a catheter tip without the need for co-registration [57]. Ramanan et al. went on to describe how catheter ablation lesions could be mapped acutely using a combination of T2 and T1* mapping with late gadolinium enhancement [58]. This has the potential for verifying the effectiveness of ablation by differentiating the actual lesion area from surrounding oedema. On a different subject, Ainslie et al. reported interim data from the multiparametric CMR assessment on RV apical versus septal pacing study (MAPS)which aims to determine the optimal site for right ventricular pacing using CMR, echo, cardiopulmonary exercise testing and biomarkers [59]. Patients with persistent atrial fibrillation requiring AV node ablation and permanent pacemaker implants were fitted with an MR-conditional St. Jude pacemaker and two RV endocardial leads placed at the RV apex and in the septum (one in the ventricular port and one in the atrial port of the device). Using a crossover study design with ventricular pacing at each of the sites, CMR measurements were taken at three time points. Early results have shown that not only is cardiac scanning safe in these patients but that there is higher aortic and pulmonary flow as well as less dyssynchrony with septal pacing.

### Congenital heart disease

CMR continues to show its superiority over other imaging modalities in the field of congenital cardiovascular disease. This year cases for one of the congenital heart case review sessions were competitively selected from open competition with blind and independent ratings from several expert reviewers. The resultant session was popular, interactive and of high quality. The benefits of CMR in congenital heart disease, not least with regards accurate anatomical delineation, were well illustrated. In addition, refinements in flow imaging are improving our understanding not only in complex congenital disease but also in more routine cases. Muzzarelli et al. presented their data on the optimal measurement of aortic flow in bicuspid aortic valve disease [60] and Hart et al. demonstrated how 4D flow can give a much greater insight into the understanding of haemodynamics of the Fontan circulation during the breathing cycle [61]. Although acquisitions can be long and complex post-processing is required, 4D flow techniques have the potential to develop into a useful clinical tool [62].

### Case presentations

As in previous years, a highly popular component of the program involved a series of 10 case review sessions, each discussing a range of interesting cases including complex, challenging problems and more mundane day-to-day cases. A lively, interactive debate was generated between delegates and experts in the field. The SCMR website publishes a regular featured ‘case of the week’ and the best 5 of these cases from 2012 were presented in full (http://scmr.org/caseoftheweek.html).

## Conclusions

In conclusion, the 2013 SCMR Scientific Sessions has consolidated a now long track record of successful meetings positioning CMR as a robust and prominent option in cardiovascular imaging. The technical advances highlighted here and the clinical applications ready to be implemented in daily routine presented at the meeting demonstrate that the method has never been so relevant and promising, with a continuous increase in the number of manuscripts published as well as a rising number of practitioners. SCMR deserves much recognition for this accomplishment and its meeting is certainly a reflection of that success.

## Competing interests

The authors declare that they have no competing interests.

## Authors’ contributions

JLF conceived the study and participated in the design, data collection and draft of the manuscript. JPC and ARP participated in the design, data collection and draft of the manuscript. All authors read and approved the final manuscript.
